# Increased P2X7 Receptor Binding Is Associated With Neuroinflammation in Acute but Not Chronic Rodent Models for Parkinson’s Disease

**DOI:** 10.3389/fnins.2019.00799

**Published:** 2019-07-31

**Authors:** Melissa Crabbé, Anke Van der Perren, Ilse Bollaerts, Savannah Kounelis, Veerle Baekelandt, Guy Bormans, Cindy Casteels, Lieve Moons, Koen Van Laere

**Affiliations:** ^1^Nuclear Medicine and Molecular Imaging, Department of Imaging and Pathology, University Hospital Leuven, KU Leuven, Leuven, Belgium; ^2^Molecular Small Animal Imaging Center, Department of Imaging and Pathology, KU Leuven, Leuven, Belgium; ^3^Laboratory for Neurobiology and Gene therapy, Department of Neurosciences, KU Leuven, Leuven, Belgium; ^4^Leuven Viral Vector Core, KU Leuven, Leuven, Belgium; ^5^Laboratory of Neuronal Circuit Development and Regeneration, Department of Biology, KU Leuven, Leuven, Belgium; ^6^Leuven Brain Institute, KU Leuven, Leuven, Belgium; ^7^Radiopharmaceutical Research, Department of Pharmaceutical and Pharmacological Sciences, KU Leuven, Leuven, Belgium

**Keywords:** Parkinson’s disease, P2X7, translocator protein, 6-OHDA, α-synuclein

## Abstract

The purinergic P2X7 receptor is a key mediator in (neuro)inflammation, a process that is associated with neurodegeneration and excitotoxicity in Parkinson’s disease (PD). Recently, P2X7 imaging has become possible with [^11^C]JNJ-(54173)717. We investigated P2X7 availability, in comparison with availability of the translocator protein (TSPO), in two well-characterized rat models of PD using *in vitro* autoradiography at multiple time points throughout the disease progression. Rats received either a unilateral injection with 6-hydroxydopamine (6-OHDA) in the striatum, or with recombinant adeno-associated viral vector overexpressing human A53T alpha-synuclein (α-SYN) in the substantia nigra. Transverse cryosections were incubated with [^11^C]JNJ-717 for P2X7 or [^18^F]DPA-714 for TSPO. [^11^C]JNJ-717 binding ratios were transiently elevated in the striatum of 6-OHDA rats at day 14–28 post-injection, with peak P2X7 binding at day 14. This largely coincided with the time course of striatal [^18^F]DPA-714 binding which was elevated at day 7–21, with peak TSPO binding at day 7. Increased P2X7 availability co-localized with microglial, but not astrocyte or neuronal markers. In the chronic α-SYN model, no significant differences were found in P2X7 binding, although *in vitro* TSPO overexpression was reported previously. This first study showed an increased P2X7 availability in the acute PD model in a time window corresponding with elevated TSPO binding and motor behavior changes. In contrast, the dynamics of TSPO and P2X7 were divergent in the chronic α-SYN model where no P2X7 changes were detectable. Overall, extended P2X7 phenotyping is warranted prior to implementation of P2X7 imaging for monitoring of neuroinflammation.

## Introduction

An increasing amount of evidence points to a pivotal role of neuroinflammation in the neuropathology of several neurodegenerative disorders, including Parkinson’s disease (PD) (Jacobs et al., 2012; Ransohoff, 2016). In the central nervous system (CNS), resident immune cells such as microglia are the main effectors of inflammation. Upon neuronal insults or infection, they become activated, directed toward a pro-inflammatory (M1-like) or anti-inflammatory (M2-like) phenotype (Cherry et al., 2014). Exacerbated inflammation and concomitant neurodegeneration have been associated with a pro-inflammatory phenotype in PD, though previous research has produced conflicting results, and more research is necessary to decipher the inflammatory state throughout the disease progression (Chung et al., 2009; Harms et al., 2018).

To date, the translocator protein (TSPO) has been the most widely used marker to image neuroinflammation with positron emission tomography (PET) and has provided us with valuable insight on the inflammatory status in PD *in vivo*. However, TSPO polymorphisms alter the binding affinity of several of the latest generation radioligands in humans, necessitating the use of genotyping (Owen et al., 2012).

In search for better imaging targets, the purinergic P2X7 receptor was put forward as a potential candidate. P2X7 is a non-selective cation channel that is expressed on CNS immune cells and plays a pivotal role in the inflammatory process (Di Virgilio et al., 2017). Its activation is sufficient to initiate the inflammatory cascade, leading to the creation of the inflammasome and subsequent IL-1β release (Karmakar et al., 2016). *In vivo* PET studies showed elevated P2X7 expression in lipopolysaccharide (LPS) models, which was confirmed by *in vitro* and *in vivo* experiments (Fantoni et al., 2017; Territo et al., 2017; Berdyyeva et al., 2019). Moreover, *ex vivo* research in animal models for Alzheimer’s disease (AD), showed a correlation between pro-inflammatory cytokines, upregulated P2X7 expression and increased ATP sensitivity, suggesting P2X7 as a potential target for therapeutic interventions countering neuroinflammation (Parvathenani et al., 2003; Sanz et al., 2009). To our knowledge, no preclinical studies on P2X7 protein expression have been performed so far in PD models.

One of the promising recently developed radiotracers is [^11^C]JNJ-54173717 (abbreviated as [^11^C]JNJ-717), which has nanomolar affinity for rat and human P2X7 and was validated both *in vivo* and *in vitro* with little interspecies differences, as compared to other radioligands (Ory et al., 2016).

To better understand the relationship between P2X7 and neuroinflammation in PD, we employed two well-characterized models: the acute 6-hydroxydopamine (6-OHDA) toxin-induced model and the more progressive recombinant adeno-associated viral vector (rAAV2/7) model overexpressing A53T α-synuclein, which translates both the PD symptoms and histopathology (Van der Perren et al., 2015b). Both models were investigated using [^11^C]JNJ-717 *in vitro* autoradiography (ARX) throughout the disease progression. In addition, P2X7 alterations were compared to the TSPO binding changes, using [^18^F]DPA-714.

## Materials and Methods

### Study Design

In this study, we evaluated the effect of the 6-OHDA toxin and viral vector-mediated A53T α-synuclein overexpression on P2X7 availability, in relation to the TSPO profile, in the nigrostriatal pathway over time.

In the toxin model, rats were subjected to unilateral 6-OHDA injections into the right striatum and acute effects on motor performance were assessed periodically. Subsequently, rats were sacrificed at pre-determined time points (4, 7, 14, 21, and 28 days) for *in vitro* ARX.

In the overexpression model, rats received stereotactic injections of recombinant adeno-associated viral vector (rAAV2/7) encoding human A53T α-synuclein or enhanced green fluorescent protein (eGFP) into the unilateral substantia nigra (SN, right hemisphere). In conjunction with behavioral analysis, rats were sacrificed at 4 days, 2, 4, 6, and 9 weeks post-injection, based on previous analysis of disease progression and *in vivo* [^18^F]FDG PET studies (Crabbe et al., 2019). Respective controls were included for every group and time point.

### 6-OHDA and rAAV2/7 A53T α-Synuclein Rat Model

All animal experiments were executed in accordance with the European Communities Council Directive 2010/63/EU and approved by the local Animal Ethics Committee of the KU Leuven (P088/2017). Female Wistar rats were injected with 6-OHDA or rAAV2/7 overexpressing α-synuclein harboring the A53T mutation (*n* = 4–7 rats/time point). An equal number of control rats were injected with, respectively, ascorbate saline or rAAV2/7 expressing eGFP. Rats were on average 8 weeks old, with a body weight range of 192–256 g. Animals had free access to pellet food and tap water, and were under a 12 h light/dark cycle.

Vector production and purification, including stereotactic injections into the SN pars compacta (SN_c_) were executed as previously described (Van der Perren et al., 2015b). In short, animals were injected with 3 μl rAAV2/7 encoding α-synuclein A53T (α-SYN) or eGFP (9.0 E + 11 genome copies (GC)/mL vector dose). The following coordinates for the SN_c_ were used: anteroposterior (AP) -5.3, lateral (LAT) -2.0, dorsoventral (DV) -7.2. For the 6-OHDA group, 24 μg of the toxin (6-hydroxydopamine hydrochloride, 28094-15-7, Merck KGaA, Germany) was dissolved in 4 μL of 0.05% ascorbate saline prior to being injected using the following coordinates for the striatum: AP: +0.2, LAT: -2.8, DV: -4.5. rAAV2/7 overexpressing the human P2X7 protein was injected in the striatum and used as a positive control, as reported previously (Ory et al., 2016; Janssen et al., 2018).

### *In vitro* [^11^C]JNJ-717 and [^18^F]DPA-714 Autoradiography

Rats were sacrificed at predetermined time points after viral vector/toxin injection, the cerebrum was excised, rapidly frozen in 2-methylbutane (-30°C until -40°C), and stored at -80°C until further processing. After cryosectioning, 20 μm transversal brain sections were mounted on glass slides and again stored at -80°C. *In vitro* ARX was executed following the protocol described by Ory et al. (2015, 2016). In short, after preincubation, brain slices were incubated for 20 min with 6 μCi/slice of [^11^C]JNJ-717 tracer solution. For [^18^F]DPA-714 this was 10 min with 16 μCi/slice. Non-specific binding was assessed on consecutive sections in conjugation with 10 μM A-740003 or 20 μM PK-11195 (861393-28-4 and 85532-75-8, Merck) as a blocking agent. Following washing steps, slides were exposed to a phosphor storage screen to obtain autoradiograms. Radioactivity concentration in the autoradiograms is indicated as digital light units (DLU) per square millimeter (mm^2^). Region-of-interest (ROI) analyses of the obtained autoradiograms were performed to quantify P2X7/TSPO binding in the SN and striatum. ROIs were manually delineated based on anatomical landmarks obtained from the Paxinos stereotactic atlas (such as the hippocampus, corpus callosum, lateral ventricles, and internal capsule for the SN). A minimum of four transverse slices throughout the ROI were included per animal and mean ligand binding minus background binding was calculated. In our analyses, the contralateral ROIs served as a reference region, in accordance with previous research (Maia et al., 2012; Wang et al., 2014; Ory et al., 2015). Therefore, data are represented as binding ratios of the DLU/mm^2^ in the ipsi- to contralateral side.

Sections from the α-SYN group belong to the same cohort as reported in a previous [^18^F]DPA-714 ARX study (Crabbe et al., 2019). Therefore [^18^F]DPA-714 results of α-SYN rats were not re-analyzed in this study.

### Behavioral Testing: Limb-Use Asymmetry Test

The cylinder test was performed to assess the symmetry of spontaneous forelimb use during explorative activity. Rats were allowed to explore a transparent glass cylinder (20 cm diameter) for 5 min, which was videotaped for analysis. The number of wall contacts by either left or right limb were counted per rat until a minimum of 20 contacts was recorded. Simultaneous contacts with both limbs were excluded and only supporting contacts were counted. Wall contacts were expressed as percentage wall contacts of the affected forelimb relative to the total number of contacts.

### Visualization of P2X7 Immunoreactivity

An additional cohort of female Wistar rats (6-OHDA/ α-SYN: *n* = 8; saline/eGFP: *n* = 8) was included for immunofluoresence staining to investigate P2X7 distribution on neurons, astrocytes, and microglia.

Rats were sacrificed using a sodium pentobarbital overdose (60 mg/kg, i.p., Nembutal, Ceva Santé Animale, Belgium) after which intracardial perfusion was performed with phosphate-buffered saline (PBS), followed by 4% paraformaldehyde in PBS. After 12 h post-fixation, samples were stored at 4°C until further processing.

For fluorescent double staining, 50 μm vibratome sections were rinsed two times in PBS with 0.2% Triton X-100, followed by a 120 min blocking step in PBS with 10% donkey serum, 0.1% bovine serum albumin (BSA) and 0.05% Triton X-100. Then, sections were incubated overnight in PBS with 0.05% triton X-100, 10% donkey serum and 0.05% BSA, including the following antibodies: goat anti-Iba-1 (polyclonal 1:500, ab107159, Abcam, United Kingdom), mouse anti-NeuN (1:500, MAB377, Merck-Millipore), mouse anti-GFAP (1:500, G3893, Merck), rabbit anti-TSPO (1:500, ab109497, Abcam) and rabbit anti-P2X7 (1:400, APR-004, Alomone Labs, Israel). Following three rinses in PBS with 0.2% triton X-100, sections were incubated for 2 h in the dark with fluorochrome-conjugated secondary antibodies: donkey anti-goat Alexa 594/680 (1:400, Molecular Probes, Invitrogen, Belgium), donkey anti-mouse Alexa 568/647 (1:400, Invitrogen), donkey anti-rabbit Alexa 488 (1:400, A-21206, Invitrogen), followed by counterstaining with DAPI (1:1000) and mounting with mowiol. The P2X7 staining was visualized using an Olympus FV1000 confocal microscope using a 60× objective and 40× for TSPO. Image contrast was adjusted using the Fiji processing package to improve image quality (Schindelin et al., 2012). For semi-quantitative analysis, a minimum of four corresponding slices per animal (*n* = 3–4 animals/group) throughout the lesion core were included to determine the percentage of P2X7-positive area by densitometry using Fiji. Images were acquired using identical exposure/gain settings.

### General Statistics

Statistics were carried out using Graphpad Prism 7 (Graphpad Software, United States). Behavioral outcomes were analyzed using the 2-way repeated measures ANOVA, with non-repeated measures ANOVA for ARX readouts. Sidak *post hoc* tests were included for correction of multiple comparisons. *P*-values < 0.05 were accepted as statistically significant.

## Results

### 6-OHDA Lesioning Is Marked by a Time-Dependent Increase in P2X7 and TSPO Binding in the Nigrastriatal Regions

The *in vitro* [^11^C]JNJ-717 regional brain binding pattern was in accordance with previous observations, showing displaceable binding in the superior colliculus, cortex, striatum, but also white matter structures such as the corpus callosum (Ory et al., 2016). For [^11^C]JNJ-717 ARX, we employed rAAV2/7-mediated hP2X7 overexpression in the striatum as a positive control (*n* = 4; ipsi- to contralateral binding ratio: 2.47 ± 0.69).

First, we assessed P2X7 availability in comparison with TSPO in an acute toxin PD model, characterized by acute nigral cell loss and striatal deafferentiation.

**FIGURE 1:**
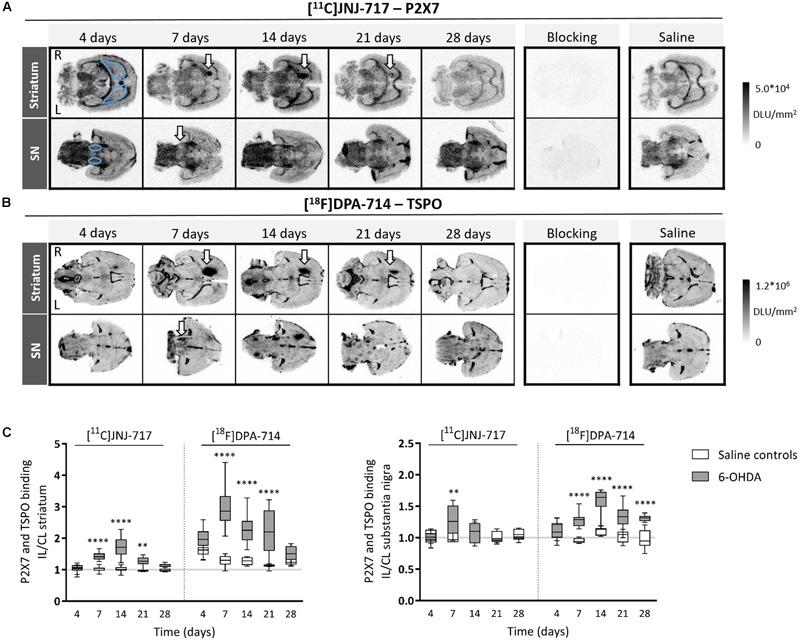
6-OHDA lesioning leads to increased P2X7 and TSPO availability in the nigrostriatal regions. **(A,B)** Representative images of [^11^C]JNJ-717 **(A)** and [^18^F]DPA-714 **(B)** autoradiograms covering the striatum and SN of 6-OHDA rats across all time points. Images of blocking studies and saline controls were included at the time point of the peak binding. Blue delineations mark the region-of-interest (ROI). Areas with increased tracer binding are indicated with white arrows. **(C)** Box plots representing ipsi- to contralateral ratios of tracer binding in the ROI. The gray lines indicate the theoretical ipsi- to contralateral binding ratio in healthy rats (∼1). *N* = 4–7 per group and per time point with ≥4 analyzed brain slices per animal. ^∗∗^*p* < 0.01, ^∗∗∗∗^*p* < 0.0001. 2-way ANOVA with Sidak *post hoc* test. 6-OHDA, 6-hydroxydopamine; IL, ipsilateral; CL, contralateral; DLU, digital light units; L, left; R, right; SN, substantia nigra.

When investigating P2X7 receptor availability, we found significantly elevated striatal [^11^C]JNJ-717 binding ratios between 7 and 21 days post-injection, in comparison with saline controls (*p* < 0.01; Figure 1A,C). Peak binding was observed at 14 days post-injection, approximately 1 week after the maximal TSPO binding ratio, albeit the [^18^F]DPA-714 peak binding was much more pronounced as compared to [^11^C]JNJ-717 (ipsi- to contralateral binding ratio: 1.01 ± 0.09 vs. 1.72 ± 0.29; *p* < 0.0001). Striatal deafferentation was associated with a subtle, but significant elevation in [^11^C]JNJ-717 binding in the affected SN at day 7 (0.99 ± 0.09 vs. 1.29 ± 0.23; *p* = 0.01; Figure 1A,C).

Striatal [^18^F]DPA-714 affected-to-non-affected-side binding ratios were significantly higher between 7 and 21 days following 6-OHDA lesioning (*p* < 0.0001; Figure 1B,C). Maximal TSPO peak binding occurred at around day 7 (1.27 ± 0.17 vs. 2.98 ± 0.62). Nigral TSPO binding was continuously elevated in the 6-OHDA group from day 7 until day 28 and reached maximal binding at day 14 (1.08 ± 0.10 vs. 1.59 ± 0.18; day 7–28: *p* < 0.001; Figure 1B,C).

### rAAV2/7-Based A53T Mutant α-Synuclein Overexpression Leads to Elevated TSPO, but Not P2X7, Binding in the Substantia Nigra

**FIGURE 2:**
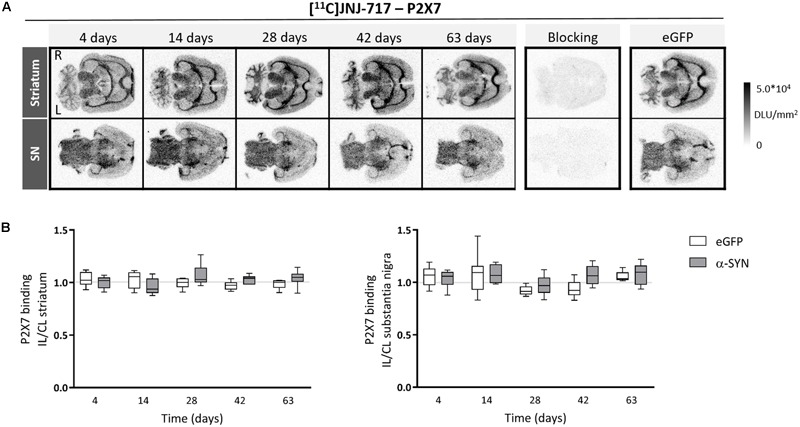
Viral vector-mediated A53T α-synuclein overexpression in the substantia nigra does not affect P2X7 availability. **(A)** Representative images of [^11^C]JNJ-717 autoradiograms across all time points obtained from rats injected with viral vector overexpressing A53T mutant α-synuclein in the substantia nigra. Images of respective blocking studies and eGFP controls at the 4-week time point were included. **(B)** Ipsi- to contralateral ratios of tracer binding in the regions-of-interest are represented in box plots. No significant changes were found. The gray lines indicate the theoretical ipsi- to contralateral binding ratio in healthy rats (∼1). *N* = 4–7 per group and per time point with ≥4 analyzed brain slices per animal. 2-way ANOVA with Sidak *post hoc* test. IL, ipsilateral; CL, contralateral; DLU, digital light units; L, left; R, right; eGFP, enhanced green fluorescent protein; α-SYN, α-synuclein.

Second, we applied our imaging strategy on the more chronic viral vector-based α-SYN model, by applying a medium dose vector titer. Nigral delivery of A53T mutant α-synuclein did not cause any changes in P2X7 availability in the striatum or SN (2-way ANOVA, *p* > 0.05; Figure 2). Significantly elevated [^18^F]DPA-714 binding ratios were present between day 28 and 42 in the SN of α-SYN rats from the same cohort (data not shown).

### 6-OHDA and α-SYN Lesioning Significantly Affects Motor Performance in Rats

**FIGURE 3:**
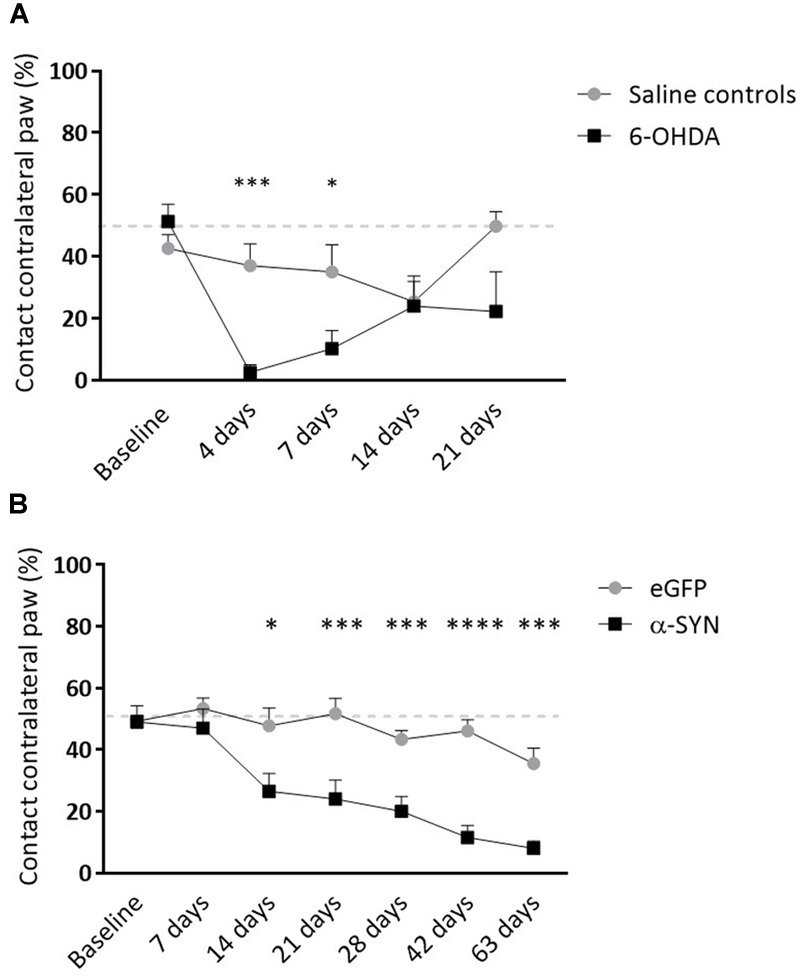
Longitudinal follow-up of motor impairment in viral vector and toxin-based models. **(A)** Striatal 6-OHDA injection is associated with partial lesioning of the motor pathway. Both groups exhibit recovery of motor impairment over time. **(B)** Nigrostriatal α-synucleinopathy leads to significant limb-use asymmetry progressively deteriorating from 2 weeks post-injection. Data are represented as mean ± SEM. ^∗^*p* < 0.05, ^∗∗∗^*p* < 0.001, ^∗∗∗∗^*p* < 0.0001, 2-way repeated measures ANOVA with Sidak *post hoc* tests.

6-OHDA rats presented with unilaterally impaired forepaw use until 7 days following surgery, after which motor performance was partially recovered (4 days: *p* = 0.0002, 7 days: *p* = 0.04; Figure 3A). As expected, α-SYN rats showed a marked decrease in the spontaneous use of the affected forepaw starting from 14 days after injection of the viral vector, continuously decreasing in the following period (% contact with contralateral paw at final time point: 35.56 ± 15.10 vs. 8.00 ± 7.89, *p* < 0.0001; Figure 3B).

### P2X7 Immunostaining Co-localizes With Iba-1-Positive Cells in the Affected Striatum of 6-OHDA Rats

**FIGURE 4:**
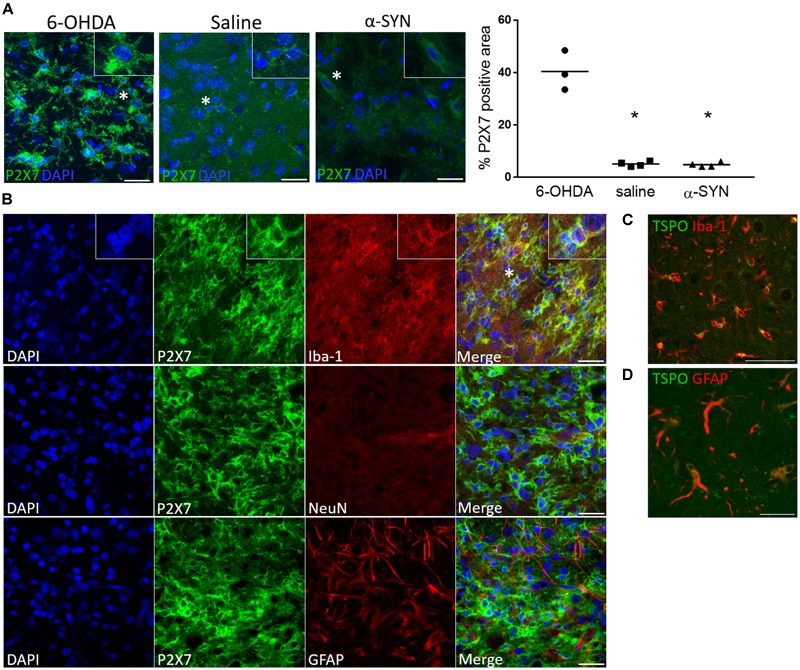
P2X7 protein expression is co-localized with Iba-1 microglial immunoreactivity following 6-OHDA-induced neuroinflammation. **(A)** A visibly lower P2X7-positive area is shown at the site of injection in the striatum of saline-injected rats at day 14 and the substantia nigra of α-SYN-rats at day 28 post-injection, as compared to the 6-OHDA model. The insets show a magnified image of the cells marked with a star. Scale bar: 25 μm. ^∗^*p* < 0.05 Mann–Whitney *U* test. **(B)** Representative details of the striatal 6-OHDA lesion core, where increased [^11^C]JNJ-717 binding was detected at day 14. P2X7 immunoreactivity is co-localized with Iba-1-positive cells of microglia/macrophage lineage, whereas the staining pattern does not coincide with GFAP or NeuN, astrocytic or neuronal markers, respectively. Scale bar: 25 μm. **(C,D)** A TSPO-positive signal is shown both in microglia and astrocytes. Scale bar: 50 **(C)** and 35 μm **(D)**.

To confirm an increased P2X7-positive cell signal, we compared the P2X7-positive cell area between 6-OHDA, saline (both 14 days post-injection) and α-SYN groups (28 days time point). A clear increase in P2X7-positive area was found in the 6-OHDA lesion (Figure 4A) whereas saline and α-SYN slices depicted a visibly lower signal intensity and density. Moreover, in the 6-OHDA striatal lesion core, where peak [^11^C]JNJ-717 binding ratios were observed, P2X7 immunostaining was unambiguously co-localized with Iba-1 reactivity (Figure 4B, top row), but not with GFAP or NeuN, indicating that peak [^11^C]JNJ-717 binding at 14 days originated from (activated) microglia. In contrast, TSPO-positive cells were of microglial (Figure 4C) and, to a lesser extent, of astrocyte origin (Figure 4D).

## Discussion

In this study, we have investigated P2X7 and TSPO availability in both an acute and progressive animal model for PD, using *in vitro* ARX. Specifically for [^11^C]JNJ-717, we found that striatal 6-OHDA lesioning was associated with higher ipsi-to-contralateral P2X7 binding, while this effect was absent in rats injected with A53T α-synuclein rAAV2/7. Both models did show significantly elevated TSPO binding in the affected brain regions.

When looking at the P2X7 binding pattern, Janssen et al. (2018) showed a similar pattern in the brains of healthy rats, and in general a higher [^11^C]SMW139 binding was observed in white matter compared with gray matter. P2X7 expression was reported in white matter structures, that may originate from immune cells such as microglia, but this is still under debate (Amadio et al., 2007; Matute, 2011). Several groups have claimed the presence of functional P2X7 in both neurons and astrocytes (Miras-Portugal et al., 2017; Munoz et al., 2017; Martin et al., 2018). In our work, P2X7 immunoreactivity clearly co-localized with Iba-1, a microglial/macrophage-specific marker, but not with neuronal or astrocyte markers. By contrast, we did not observe P2X7 protein co-localization with neuronal or astrocyte markers subsequent to striatal 6-OHDA lesioning.

In previous studies on neuroinflammation and P2X7, systemic LPS injections were associated with an elevated whole-brain distribution volume of [^11^C]GSK1482160 at 72 h post-injection, though this effect was less pronounced with [^18^F]EFB at 24 h (Fantoni et al., 2017; Territo et al., 2017). Following a 20 μg LPS striatal injection, Berdyyeva et al. (2019) reported a 31% increase in [^18^F]JNJ-64413739 SUV values at 2 days post injection, which was confirmed by *ex vivo* ARX.

In more progressive neuropathologies, such as AD, both increased and decreased P2X7 expression was reported, by use of immunohistochemistry and [^123^I]TZ6019 *in vitro* ARX (Parvathenani et al., 2003; McLarnon et al., 2006; Janssen et al., 2018; Jin et al., 2018). Interestingly, immunohistochemical staining indicated increased P2X7 levels in AD gray and white matter tissue, together with other neuroinflammatory markers (MHC-II, CD68, Iba-1), but this was not observed using [^11^C]SMW139 ARX (Janssen et al., 2018). This discrepancy could be explained by the limited spatial resolution of ARX (micrometer range) as compared to immunohistochemistry (nanometer range), and is also a limitation in our study. Despite the absence of possible receptor up- or downregulations, P2X7 KO mice showed reduced Aβ lesions, improved cognitive function and synaptic plasticity (Martin et al., 2018).

Since P2X7 is activated by micro- to millimolar levels of ATP (normal ∼ nanomolar range), it is plausible that P2X7 expression remains relatively stable in diseases with a slow progression and a non-focal inflammatory pattern, such as several neurodegenerative disorders (Wilhelm et al., 2010). Nevertheless, P2X7 receptor function might significantly impact the pathogenesis of these pathologies and continues to be a valuable target for treatment of various inflammatory and infectious diseases because of its link with both innate and adaptive immune responses (Savio et al., 2018).

In this project, we aimed to compare the timeline of P2X7 changes to the TSPO. Whereas a time course coincidence was found between P2X7 and TSPO overexpression in the acute 6-OHDA model, we did not observe this in the more chronic A53T α-SYN model.

These results are in line with the TSPO timeline shown by Maia et al. (2012) using [^125^I]CLINDE ARX in 6-OHDA rats. Increased TSPO expression has been associated with pro-inflammatory signaling of TNF-α and iNOS in activated microglia (Beckers et al., 2018). Similarly, P2X7 stimulates the release of pro-inflammatory factors, such as IL-6 from human fibroblasts, TNF-α from mouse microglia (Di Virgilio et al., 2017). Alternatively, P2X7 stimulation in macrophages was able to release anti-inflammatory proteins, such as Annexin A1, independent of the microglial inflammatory phenotype (de Torre-Minguela et al., 2016). In our study, the divergence between the TSPO and P2X7 profile might indicate that P2X7 is a marker of an alternate inflammatory state. Altogether, these findings underline the therapeutic potential of treatments targeting the immune response in neurodegenerative disease. Although immunohistochemical analysis suggest that P2X7 expression is increased in the 6-OHDA model, we cannot definitively conclude whether elevated striatal [^11^C]JNJ-717 binding is caused by an increased cellular density of P2X7-positive microglia rather than increased protein expression or altered microglia morphology. Histology confirmed a markedly increased P2X7-positive cell area. Therefore, future studies should focus on the relationship between microglial P2X7 function and PD pathogenesis, quantified by both *in vivo* and *in vitro* readouts.

We previously demonstrated that α-synucleinopathy is associated with higher displaceable [^18^F]DPA-714 binding in the unilateral SN, from 28 to 42 days into the disease pathology (Crabbe et al., 2019). TSPO overexpression was shown to predominantly mark activated microglia of the pro-inflammatory phenotype (Beckers et al., 2018). In the α-SYN model, a clear microglial activation and increased CD68 and MHC II-positive cell density was reported in the affected SN at 29 days post-injection, together with elevated CD4- and CD8-positive T-cell infiltration (Van der Perren et al., 2015a). Evidence from [^18^F]DPA-714 ARX would suggest that a general pro-inflammatory phenotype is present around 28–42 days post-injection. However, due to the progressive nature of the α-SYN model, it is possible that the P2X7 expression pattern marks a different acute inflammatory state. We also cannot exclude that affinity of [^11^C]JNJ-717 (IC_50_∼7.6 nM) for the rat P2X7 protein is too low to detect small changes in microglial P2X7 availability. We utilized *in vitro* ARX instead of *in vivo* imaging since this technique has higher sensitivity, spatial resolution and higher throughput per tracer batch, which renders it particularly suitable to explore possible P2X7 alterations. Alternatively, P2X7 may mediate pro-inflammatory processes outside of the investigated time window, as 2-week intervals were present between measurements.

As expected, rats from the α-SYN group showed a behavioral deficit starting from 14 days post-injection, resulting in a >80% decrease in use of the contralateral forelimb. In accordance with previous reports, this corresponds to a pronounced ∼80% loss of dopaminergic neurons in the nigrostriatal pathway (Van der Perren et al., 2015b). A single injection of 24 μg 6-OHDA into the right striatum resulted in progressive dopaminergic lesioning with partial recovery of behavioral deficit between 4 and 14 days following surgery. Indeed, a single 6-OHDA injection causes localized degeneration of dopaminergic terminals in the striatum whereas three or four injection sites are necessary to induce a near complete dopaminergic depletion (Decressac et al., 2012). Behavioral recovery is a well-established phenomenon following partial 6-OHDA lesioning and is thought to originate from axonal sprouting of surviving dopaminergic neurons (Dravid et al., 1984; Finkelstein et al., 2000; Arkadir et al., 2014).

Together, our findings show for the first time an increased microglial P2X7 availability in the acute 6-OHDA model for PD, in parallel with the time course of regional TSPO changes. We confirmed that strong P2X7 immunoreactivity was present on microglia in the 6-OHDA lesion, but not astrocytes or neurons. In the rAAV2/7 α-synuclein overexpression model, we could not demonstrate increased P2X7 availability with [^11^C]JNJ-717. This suggests that an alternative immune activation might occur as a response to α-synucleinopathy, where altered P2X7 protein expression might not take place. Further research is warranted to confirm the microglial P2X7 phenotype and to better comprehend its role in pathogenesis of neurodegenerative diseases.

## Data Availability

The raw data supporting the conclusions of this manuscript will be made available by the authors, without undue reservation, to any qualified researcher.

## Ethics Statement

All animal experiments were executed in accordance with the European Communities Council Directive 2010/63/EU and approved by the local Animal Ethics Committee of the KU Leuven (P088/2017).

## Author Contributions

MC, AVdP, LM, and KVL contributed to the conception and design of the study. MC and SK performed the autoradiography experiments. MC and IB performed the immunohistology. MC performed the statistical analysis and wrote the first draft of the manuscript. GB, VB, LM, and KVL provided the funding and hardware needed for this study. All authors contributed to the manuscript revision, and read and approved the submitted version of the manuscript.

## Conflict of Interest Statement

The authors declare that the research was conducted in the absence of any commercial or financial relationships that could be construed as a potential conflict of interest.
